# Interventions targeting the gut microbiota and their possible effect on gastrointestinal and neurobehavioral symptoms in autism spectrum disorder

**DOI:** 10.1080/19490976.2025.2499580

**Published:** 2025-05-16

**Authors:** Evelyn Takyi, Khemlal Nirmalkar, James Adams, Rosa Krajmalnik-Brown

**Affiliations:** a Biodesign Center for Health Through Microbiomes, Arizona State University, Tempe, AZ, USA; b School for Engineering of Matter, Transport, and Energy, Arizona State University, Tempe, AZ, USA; c School of Sustainable Engineering and the Built Environment, Arizona State University, Tempe, AZ, USA

**Keywords:** Autism, ASD, gut microbiome, fecal microbiota transplant, FMT, microbiota transplant therapy, MTT, prebiotics, probiotics, synbiotics

## Abstract

Autism spectrum disorder (ASD) is a developmental disorder that is characterized by deficits in social communication and restricted, repetitive, and stereotyped behaviors. In addition to neurobehavioral symptoms, children with ASD often have gastrointestinal symptoms (e.g. constipation, diarrhea, gas, abdominal pain, reflux). Several studies have proposed the role of gut microbiota and metabolic disorders in gastrointestinal symptoms and neurodevelopmental dysfunction in ASD patients; these results offer promising avenues for novel treatments of this disorder. Interventions targeting the gut microbiota – such as fecal microbiota transplant (FMT), microbiota transplant therapy (MTT), probiotics, prebiotics, synbiotics, antibiotics, antifungals, and diet – promise to improve gut health and can potentially improve neurological symptoms. The modulation of the gut microbiota using MTT in ASD has shown beneficial and long-term effects on GI symptoms and core symptoms of autism. Also, the modulation of the gut microbiota to resemble that of typically developing individuals seems to be the most promising intervention. As most of the studies carried out with MTT are open-label studies, more extensive double-blinded randomized control trials are needed to confirm the efficacy of MTT as a therapeutic option for ASD. This review examines the current clinical research evidence for the use of interventions that target the microbiome – such as antibiotics, antifungals, probiotics/prebiotics, synbiotics, and MTT – and their effectiveness in changing the gut microbiota and improving gastrointestinal and neurobehavioral symptoms in ASD.

## Introduction

Autism spectrum disorder (ASD) is a multifactorial neurodevelopmental disorder characterized by verbal communication deficiency, social interaction impairment, restricted interests, and repetitive behaviors.^1^ It affects about 1 in 36 children in the United States,^2^ impacting families and impeding the developmental progress of affected children.^3^ While ASD etiology is unclear, a combination of several factors, such as genetic and environmental,^4,5^ immune dysregulation, inflammation,^6^ and microbiome imbalances,^7^ may play a role in the onset and development of ASD. Individuals with ASD experience symptoms that vary in severity, and there are many common co-morbid symptoms in ASD,^8^ including gastrointestinal symptoms,^9^ such as abdominal pain, diarrhea, constipation, gas, reflux, bloating, and vomiting. Some studies have found an association between gastrointestinal (GI) symptoms and the severity of ASD-related symptoms.^10–16^ ASD and GI issues may be linked to dysbiosis in the gut microbiota through the microbiota-gut-brain axis, a bidirectional connection between the microbiota, the gut, and the brain.^17,18^ Many studies have reported differences in the composition of the gut microbiota between ASD patients and neurotypical individuals.^19–36^ Some of the differences can be attributed to (1) excessive use of antibiotics by ASD individuals,^37,38^ which can influence gut homeostasis by targeting pathogens and commensal bacteria;^39,40^ (2) dietary restrictions as children with ASD have unique dietary preferences;^41^ (3) host genetics;^42^ (4) mode of delivery;^43^ and (5) feeding patterns,^44^ including breastfeeding or formula feeding and/or supplementation.

The increasing rates of diagnoses and a lack of effective therapeutic options highlight the importance of investigating alternative treatments for ASD. A growing number of studies explored the potential impact of microbiota-based interventions in animal and human studies. ASD studies involving animal models that used probiotics^45,46^ and fecal microbiota transplant (FMT)^47–51^ showed positive effects by relieving the ASD-like behaviors in mouse models. For instance, the treatment with *Bacteroides fragilis* reduced gut permeability, altered gut microbiota composition, and decreased ASD-like behaviors in a mouse model of ASD.^45^ Some of the findings from these animal studies also support the evidence of human studies described in Tables 1 and 2. Gilbert et al. summarized a path for how interventions based on microbiome profiling can be used for neurodevelopmental disorders, such as ASD, moving the knowledge from pre-clinical animal models, such as with mice, to clinical trials with humans.^52^ However, this approach has not always been used, and it is often not needed.

**Table 1.** Clinical trials of probiotic/prebiotics/synbiotic studies in ASD.

Reference	Study design	Intervention	Findings
**Probiotic Study**			
Kong et al.^142^	Randomized double-blind placebo-controlled ASD Participants: 35 Treated: 18(3.6–18.50 yrs) Placebo:17(4.69–19.70 years) Diagnosis tool: ADOS-2 and DSM-5	*Lactobacillus plantarum* PS128 probiotic (6 × 10^10^ CFUs)/day was administered for 28 weeks. Intranasal oxytocin spray was given to the treated and placebo group at week 16. Primary and secondary outcome measures were assessed at 0, 16, 28 weeks.	Improvements in SRS and ABC(primary outcome) and Clinical Global Impression (CGI) (secondary outcome) in the treatment group that received the probiotics and intranasal oxytocin spray compared to the placebo group. *Roseburia, Veillonella*, and *Streptococcus* were higher in the probiotic-treated group
Guidetti et al.^143^	A randomized, double-blind crossover study with a placebo ASD Participants: 61(24 months −16 yrs) Treated: 30 Placebo: 31 Diagnosis tool: ADOS-2 and ADI-R	10 × 10^9^ CFU of the probiotic mixture(*Limosilactobacillus fermentum* LF10, *Ligilactobacillus salivarius* LS03, *Lactiplantibacillus plantarum* LP0, *Bifidobacterium longum)* was administered to the treatment group per day for 3 months. The placebo group received 2.5 g of maltodextrin in powder form.	Improvements in GI symptoms, communication, and maladaptive behaviors. *Streptococcus thermophilus*, *Bifidobacterium longum*, *Limosilactobacillus fermentum*, and *Ligilactobacillus salivarius* species were increased in treatment group, and *Alistipes finegoldii, Clostridium leptum, and Ruminococcus calldus were decreased*
Sherman et al.^144^	Randomized placebo-controlled ASD Participants: 35(3-20 yrs) Treated: 18 Placebo: 17	The treatment group received daily *Lactobacillus plantarum* probiotic (6 × 10^10^ CFUs), and the placebo group received microcrystalline cellulose for 16 weeks.	No change in ASD severity and GI symptoms. Increase in *Lactobacillus*.
Shaaban et al.^145^	Prospective open-label study, Participants ASD: 30 (2-11 yrs) TD Controls: 30 typically developing age and gender match	5 g of daily dose of 100 × 10^6^CFU of probiotics (*Lactobacillus acidophilus, Lactobacillus rhamnosus*, and *Bifidobacteria longum*) 1 time/day for 3 months	Decrease in total ATEC scores (the primary outcome) and an improvement in the subscales of ATEC: speech, language, communication, sociability, and cognitive awareness. Improvements in GI symptoms (secondary outcome) measures: constipation, abdominal pain, flatulence. Increase in *Bifidobacteria* and *Lactobacilli* levels after supplementation
**Prebiotic study**			
Inoue et al.^155^	Open-label study, Participants ASD: 13 (4-9 yrs) TD Controls: 123 typically developing (2-75 years) Diagnosis tool: DSM-5	Prebiotic dietary supplement (partially hydrolyzed guar gum (PHGG) 6 g/day administered to ASD children and lasted 2 months or longer.	Reduction in constipation, behavioral irritability, and an increase in defecation per week in participants. *Blautia* and *Acidaminococcus* increased while *Streptococcus*, *Odoribacter*, and *Eubacterium* decreased after treatment
Grimaldi et al.^156^	Randomized, double-blind, placebo-controlled, Participants ASD: 30 (4-11 yrs) TD Controls: No controls	Children were randomly assigned to placebo and treated group using a random number system. The placebo group received maltodextrin-GLUCIDEX®; 1.8 g was given in powder form. The treated group received 1.8 g Bimuno galacto oligosaccharide (B-GOS) prebiotic supplementation during the 6-week feeding period.	Improvement in abdominal pain, bowel movements, and ASD symptoms, such as social behavior. Increase in beneficial microbes, such as *Coprococcus spp*. and *Dorea formicigenerans*.
Synbiotic study			
Li et al.^146^	Open-label study Participants ASD: 53 (3-12 yrs) TD Controls: 45 typically developing age and gender match Diagnosis tool: DSM-5	Children received dry powder of probiotic *Bifidobacterium animalis* subsp. *lactis* Probio-M8 (Probio-M8)(1.0 × 10^11^CFU/g) daily for a period of 12 weeks, with a balanced diet (40% carbohydrates, 30% fats, and 30% proteins).	Improvement in CARS scores (the primary outcome) and GI symptoms (secondary outcome) and increased levels of *Bifidobacterium animalis*, *Akkermansia muciniphila, Fusicatenibacter saccharivorans*, and *Sutterella* sp. while also reducing *Blautia obeum*.
Phan et al.^57^	Open-label Participants ASD: 170 (2-8 yrs) TD Control: 123 typically developing (2-75 years) Diagnosis tool: SRS2	ASD patients received a 3-month supplementation of personalized synbiotic.	No improvement in ASD related symptoms assessed using Social Responsiveness Scale (SRS2) (the primary outcome). However, there was an improvement in GI symptoms (secondary outcomes).The proportion of *B. breve, L. reuteri, L. plantarum*, and *L.brevis* increased in ASD patients after synbiotic treatment.
Wang et al.^147^	A double-blind, placebo-controlled, Total ASD Participants: 26(2-8 yrs). Treated: 16 and Placebo:10 TD Controls: 24 typically developing (2-8 years) Diagnosis tool: DSM-5 and CCMD-3	10^10^CFU of four probiotics (*B. infantis Bi26, L. rhamnosus HN001, B. Lactis BL-04*, and *L. Paracasei rhamnosus* HN001) and fructo-oligosaccharide (FOS) were given to the treated group for 30 days. The placebo group received maltodextrin.	Reduction in the severity of autism assessed using ATEC (primary outcome) and improvements GI symptoms measured by GSRS (secondary outcome) compared to the placebo group. Increase in beneficial bacteria, such as *Bifidobacteriales*, and a decrease in *Clostridium* after intervention.
Sanctuary et al.^148^	Randomized, double-blind crossover-controlled trial Participants ASD: 8 (2-11 yrs)	20 billion CFU per day of *Bifidobacterium infantis* and colostrum were administered for 4 weeks, followed by wash out period for 2 weeks and 5 weeks of colostrum.	Reduced aberrant behaviors and GI symptoms. No differential abundant genera were observed after treatment.
Arnold et al.^150^	Randomized placebo-controlled, crossover trial Total ASD Participants:10 (3-12 yrs) Treated: 6 Placebo: 4 Diagnosis tool: ADOS-2 and ADI-R	900 billion bacteria per packet of probiotic mix (VISBIOME), consisting of *L. casei*, *Lactobacillus plantarum*, *Lactobacillus acidophilus*, and *Lactobacillus delbrueckii* subsp. *Bulgaricus*, *B. longum*, *Bifidobacterium infantis*, and *Bifidobacterium breve*, and *S. thermophiles* and starch were administered half packet twice daily for the first 4 weeks, then increased to an entire packet twice daily for the last 4 weeks if no effects were seen.	No improvement in pediatric quality of life inventory (PedsQL) (primary outcome), but they reported a moderate effect size compared to the placebo. Improvements in GI symptoms based on parents selected target symptoms (secondary outcome) with a large effect size. There were no significant alterations in the microbiome community.
Aldegheri et al.^149^	Single patient case study Participant: 17-year-old adolescent male	The patient received 2 tablets of antibiotics, Rifaximin, three times daily for 10 days, followed by a daily intake of 12.5 billion *Bifidobacterium lactis* Bi-07 and *Lactobacillus acidophilus*, as well as 250 mg of 2’-FL fucosylated HMO for 6 months.	Improvement in aggressiveness, increased mood stability and reduction in constipation. Decrease in *Sutterella* spp. after treatment.

ADI-R: Autism Diagnostic Interview-Revised; ADOS-2: Autism Diagnostic Observation Schedule, Second Edition (ADOS-2); DSM-5: Diagnostic and Statistical Manual of Mental Disorders; CARS: Childhood Autism Rating Scale; ABC: Autism Behavior Checklist; SRS: Social Responsiveness Scale; CGI: Clinical Global Impression; CCMD-3: Chinese Classification of Mental Disorders; GSRS: Gastrointestinal Symptom Rating Scale; ATEC: Autism treatment evaluation checklist; TD: typically developing.

**Table 2.** MTT clinical trials in ASD.

Reference	Study design	Pre-MTT treatment	Intervention	Findings
Kang et al.^54^	Open-label Participants ASD: 18 (7-16 yrs) with GI symptoms Control: 20 neurotypicals (age and gender-matched) without GI symptoms Diagnosis tools: ADI-R	Antibiotic treatment: oral vancomycin for 2 weeks Proton pump inhibitor Bowel cleansing	MTT(SHGM) Initial high dose 2.5 × 10^12^cells/day for 2 days Maintenance dose 2.5 × 10^9^ cells/day for 7–8 weeks Administration route: oral and rectal. Follow-up: 8 weeks and 2 years	80% reduction in GI symptoms and 23% improvement in ASD symptoms. Improvement in GI and ASD was maintained after 2 years. Increased microbial diversity. Increased relative abundance of *Prevotella*, *Desulfovibrio*, and *Bifidobacteria*
Li et al.^172^	Open-label Participants ASD: 40 (3-17 yrs) with GI symptoms Control: 16 neurotypical (age and gender match) without GI symptoms. Diagnosis tool: ADI-R	Bowel Cleanse: Participants received 2 L Golytely (polyethylene glycol) the night before the transplantation	2×10^14^CFU once a week for 4 weeks Administration route: Oral and rectal. Follow-up: 8 weeks	35% decrease in GI symptoms and 10% decrease in CARS scores after four weeks of treatment *Eubacterium Coprostanoligene* was reduced in abundance after MTT treatment
Hu et al.^176^	Single patient case study Participant: 7-year-old female. Diagnosis tool: ADOS-2; DSM-5	Antibiotic treatment: oral vancomycin for 14 days Bowel preparation: fasting for 8 hours, water prohibition for 4hrs, and administered polyethylene glycol electrolyte powder	MTT fluid 80 ml of bacterial solution was administered five times, separated by 1 week through colonoscopy. No follow up	ATEC, SRS, and CARS scores decreased after vancomycin treatment and further decreased after MTT treatment. Gastrointestinal symptoms also improved. *Bacteroides* and *Ruminococcus* increased, and *Bifidobacterium*, *Anaerostipes*, *Streptococcus*, and *Faecalibacterium* decreased after MTT
Li et al.^174^	Open-label Participants ASD: 38 (3-14 yrs) 31 with GI symptoms and 7 with no GI symptoms Control: 30 neurotypical (age and gender match) without GI symptoms. Diagnosis tool: DSM-5	No antibiotic treatment was done	Lyophilized MTT capsules were administered orally consisting of 1 g of donor stool per 1 kg of recipient body weight, once every 4 weeks for a total of 12 weeks 8 weeks follow-up	At the end of treatment, there was decrease in GSRS (51%), ABC (20%), CARS (10%) from baseline. At the end of follow-up, there was a decrease in GSRS (32%), ABC (23%), CARS (10%) and SRS (6%) from baseline. Increase in the abundance of *Eubacterium_hallii_group, Anaerostipes, Fusicatenibacter, Collinsella*, *Ruminococcus_torques_group* , and *Dorea*, and decrease in the abundance of *Blautia Prevotella* and *Sellimonas*
Chen et al.^175^	Open-label Participants ASD: 29 (2-11 yrs) with GI symptoms Control: 36 neurotypical (age and gender match) Diagnosis tool: DSM-5	No antibiotic treatment or bowel cleanse was done	2 capsules of freeze-dried microbiota (bacterial cells were equivalent to 200 g of fresh stool) was administered orally for 12 days per month for 4-months	Improvement in ABC, CARS, and GI symptoms after treatment A decrease in *Collinsella*
Hazan et al.^177^	Single patient case study Participant: 19-year-old male Diagnosis tool: Not stated	Antibiotic treatment: 500 mg of vancomycin three times daily Deep colonic wash prior to colonoscopy	A single dose of MTT (300 mL) was infused directly into the cecum via colonoscopy. Follow-up: 15 months	Improvement in CARS, ATEC, and GI symptoms. *Bifidobacterium* increased, and *Lactobacillus animalis* decreased after treatment

ADI-R: Autism Diagnostic Interview-Revised; DSM-5: Diagnostic and Statistical Manual of Mental Disorders; CARS: Childhood Autism Rating Scale; ABC: Autism Behavior Checklist; SRS: Social Responsiveness Scale; GSRS: Gastrointestinal Symptom Rating Scale; SHGM: standardized human gut microbiota; ATEC: Autism treatment evaluation checklist.

The above-mentioned strategies are important to test specific hypotheses for mechanistic research. However, clinical studies of microbiota-based interventions in ASD patients, although less mechanistic, provide translational evidence of the importance of the microbiota-gut-brain axis in ASD. Some of the clinical trials’ interventions – such as FMT,^53^ microbiota transplant therapy (MTT),^54^ antibiotics,^55^ probiotics,^56^ and synbiotics^57^ as novel strategies to modify the microbiome – demonstrated improved GI symptoms and core symptoms in individuals with ASD, enhancing their health and quality of life.

In this review, we focus on studies of interventions that target the microbiota to treat gastrointestinal and ASD-related symptoms and that reported changes in the microbiota of study participants after the intervention. We start with a brief description of the gut microbiota, metabolites, and immune system dysregulation associated with ASD, followed by clinical studies on interventions that target the microbiota, including antibiotics, antifungals, probiotics/synbiotics, diet/prebiotics, and FMT/MTT.

## Gut microbiota and metabolites associated with ASD

Increasing reports have pointed to the possible role of the gut microbiota in ASD pathogenicity.^21,23,25,26,32,34–36,58–68^ Despite several reports on the gut microbial imbalance in ASD, there is no defined microbial signature for ASD diagnosis.^7,69^ However, multiple studies have found a significantly lower microbial diversity^26,28,30,57^ and a greater abundance of microbes, including some in the *Clostridium* and *Bacteroides* genera, in the gut microbiota of children with ASD.^24,30,53,70–74^ Also, some studies found a lack of other fiber fermenters, such as *Prevotella*.^19,26,27,58^ Two recent meta-analyses noted a reduced presence of *Bifidobacterium* at the genus level in the gut microbiota of children with ASD.^24,69^ A meta-analysis of multiple cohorts found 591 microbes that were more common in the gut microbiome of children with ASD and 169 microbes that were less common in their control counterparts.^41^ Recent multi-kingdom metagenomic analyses in a large cohort (*n* = 1627) revealed *Virgibacillus* species were enriched and *Desulfovibrio vulgaris* and *Bacteroides* species were depleted in ASD children.^75^ Overall, these studies reveal not only large variations in the microbiome of children with ASD but also some significant differences compared to typically developing (TD) children.

The gut microbiome is highly variable among individuals. The variations may be due to several factors, which were described in Krajmalnik-Brown et al. such as the use of siblings as controls or avoiding the use of relatives as controls, large heterogeneity in the microbiomes of individuals because of differences in geographic^17^ or ethnicities, and diet^76,77^ and children with ASD generally have more restricted diets.^41^ Also, there are variations in the findings from various studies, and these may be due to variety of protocols for sampling and techniques for characterizing the microbial ecology – such as workflows for specimen storage and processing, lack of standardization of experimental protocols, different analysis methods, and the extent to which other confounding factors were considered – could account for the inconsistencies in the findings from the studies.^78–81^

Several microbial-related mechanisms have been implicated in ASD. Microbial dysbiosis induces the breakdown of the gut integrity,^82,83^ produces toxins,^58^ and results in immunological^84^ and metabolic abnormalities. Alteration in intestinal permeability (referred to as “leaky gut”) can result in the release of proinflammatory substances, such as Lipopolysaccharide (LPS), which can modulate the central nervous system by increasing activity in brain areas, such as the amygdala that controls emotions and behavior.^85^ For instance, a microbial shift within the gut of mice yielded changes in serum metabolites and induced ASD-like phenotypes.^45^

The gut microbiota is a crucial component of the gut-brain axis and plays an important role in influencing the brain through endocrine, immunological, metabolic, and neurological systems.^86,87^ The gut microbiota influences brain functions via the secretion of active metabolites – such as neurotransmitters (e.g., serotonin), short chain fatty acids (SCFA), and immune modulators – which can cross the blood-gut and the blood-brain barriers.^9,33,88^ Dysregulation in the production or consumption of these compounds can induce ASD-like behaviors by affecting the host’s immune and neural system and modulating the host’s development.^45,89,90^ The level of an excitatory neurotransmitter, glutamate, has been reported to be higher in the feces and blood of individuals with autism.^60,91^ Also, an increased ratio of glutamate to GABA is known to be a signature of neuroinflammation linked to sensory processing, and the ratio between these two major neurotransmitters was reported to be different in blood samples from children with ASD compared to those from TD children.^92^ Altered levels of other neurotransmitters (e.g., serotonin and dopamine) and amino acids (e.g., tryptophan and indoles) have also been linked to autism and regulated by the gut microbiome.^51^

Pre-clinical studies have also shown that microbial metabolites, such as para-cresol^93^ and 4-ethylphenol sulfate,^94^ can cause ASD-like symptoms in mice. Similarly, high levels of urinary p-cresol sulfate have been correlated with ASD-related symptoms in humans.^95,96^ Microbes, such as *Clostridium difficile* (now reclassified as *Clostridioides difficile*),^97^ *Blautia hydrogenotrophica*, *Romboutsia lituseburensis*, and *Anaerostipes hadrus*, produce p-cresol.^98^ Although these microbes are known to produce p-cresol, most of them have not been reported in higher abundance in ASD individuals. Only a few studies have documented a higher abundance of *Clostridium* ,^21,99–102^ and a single study has reported an association with *Anaerostipes* ^103^ in the ASD population. P-cresol has many toxic properties and it is known to affect gamma-aminobutyric acid (GABA) and glutamate transport, causing neurological and physiological changes in patients with ASD^104,105^ and reducing glutathione (GSH), which acts as an antioxidant.^106^

**Figure 1.**
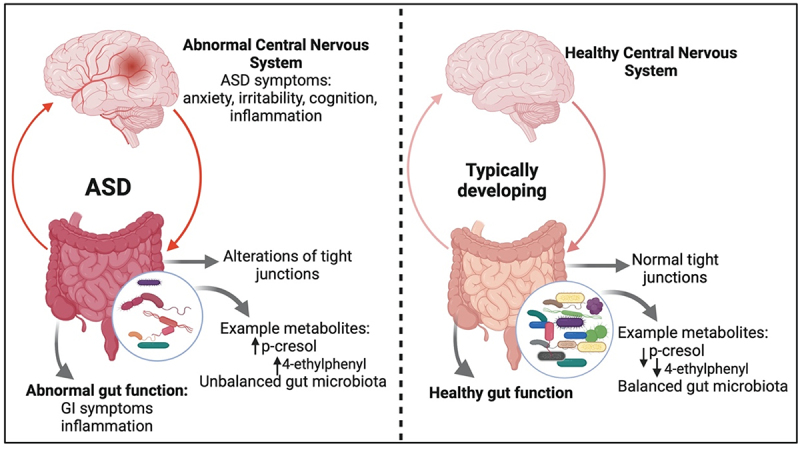
An overview of the gut microbiome effects on gastrointestinal symptoms and possible metabolite-based gut-brain connection in ASD and typically developing individuals.

This suggests that harmful microbial metabolites contribute to ASD symptoms in some children with ASD. Reviews by Peralta-Marzal et al. and Siracusano et al. reported differences in several metabolomic profiles in fecal, urine, and blood samples from individuals with ASD and typically developing controls.^107,108^ In addition, microbial species that colonize the gut in children with ASD can alter the synthesis of beneficial microbial products, such as short chain fatty acids (SCFA), vitamins, metabolites, and neurotransmitters, essential for human health and communication with the brain (Figure 1).^109,110^ For example, a meta-analysis by Morton et al. found that amino acid metabolism, carbohydrate metabolism, and lipid metabolism encoded by microbial species in the genera *Prevotella*, *Bifidobacterium*, *Desulfovibrio*, and *Bacteroides* correlated with changes in brain gene expression.^41^ Thus, abnormal gut microbiota in children with ASD provides a suitable target for microbiota-targeting interventions.

## Gut microbiota and immune system dysregulation in ASD

One important connection with the gut microbiome is the immune system. The microbiota plays a role in immune system maturation, and microbial dysbiosis in autism can lead to immune system dysregulation.^45,84,111^ The activated immune system, as a result of microbiota dysbiosis, releases chemokines and cytokines – such as interleukin-1β (IL-1β), interleukin-6 (IL-6), interferon-γ (INF-γ), tumor necrosis factor-α (TNF-α) – which cross the blood brain barrier (BBB) and these mediators can bind to endothelial cells in the brain and induce immune responses in the brain.^112–114^ A review by Critchfield et al. reported abnormal immune system function in ASD.^115^ Ashwood et al. found that proinflammatory cells such as Cluster of Differentiation 3 tumor necrosis factor-alpha (CD3+ TNFα+), Cluster of Differentiation 3 Interleukin-2 (CD3+ IL-2+), and Cluster of Differentiation 3 Interferon‐gamma(CD3+ IFNγ+), were higher in participants with ASD compared to typically developing controls.^116,117^ In contrast, mucosal Cluster of Differentiation 3 Interleukin-2 (CD3+ IL-10+) cells were lower in ASD children compared to typically developing controls. Enstrom et al. also found that immunoglobulin (IgG4) levels were higher in ASD compared to typically developing controls.^118^ A meta-analysis by Morton et al. showed that bacteria, such as *Bacteroides thetaiotaomicron*, abundance was correlated with lower transforming growth factor beta (TGF-β) levels.^41^ This bacteria is known to modulate maternal immune activation-dependent metabolites that are linked to behavioral symptoms.^45^ The authors also found that higher abundances of *Bifidobacterium longum* and *Prevotella copri* were associated with lower interleukin-6 (IL-6) (proinflammatory) levels.^41^

## Interventions to modify the microbiome

Many studies have explored the potential impact of microbiota-based approaches to restore the ecological balance in the gut affected by dysbiosis and improve GI and core symptoms in individuals with ASD. The most common ways to change the gut microbiome are antibiotics, antifungals, probiotics, synbiotics, prebiotics, diet, fecal microbiota transplant, and microbiota transplant therapy.^119,120^ Some of these approaches are promising not only in the treatment of GI symptoms among people with ASD but also in alleviating other ASD-related symptoms.

### Antibiotics

Antibiotics alter the gut microbial balance by suppressing the growth of many gut bacteria, including beneficial ones.^121,122^ Vargason et al. found that early exposure to antibiotics may result in a rise in recurring GI symptoms,^16^ suggesting that they may contribute to GI and possibly ASD symptoms by disrupting the gut microbiota. Many other studies have reported increased antibiotic use in children with autism versus typically developing children, especially for treating ear infections.^38,123,124^ Studies have shown that long-term antibiotic exposure during pregnancy or early infancy has been associated with ASD.^125,126^ The increased use likely increased their risk of developing gut microbiota imbalances, and possible enrichment of antibiotic-resistance genes in the microbiota, which may increase the individual’s vulnerability to infections^127^ leading to recurring GI disorders.^37^

A survey study of over 27,000 autism families reported that antibiotics were much more likely to result in the worsening of symptoms (33%) than improving symptoms (18%), with 2507 families reported.^128^ However, a clinical trial by Sandler et al.^55^ showed that oral vancomycin treatment for 8 weeks (much longer than the standard 10 days) resulted in temporary beneficial changes in GI and ASD symptoms. However, most improvements were lost within a few weeks when the treatment stopped. Oral vancomycin acts only on gut bacteria because it has minimal absorption into the rest of the body, therefore, strongly suggesting that alterations in the gut bacteria were the primary cause of the GI symptoms and contributed substantially to ASD symptoms. However, the temporary nature of the benefits suggests that harmful bacteria quickly regrew after treatment stopped. Similarly, a case series reported by Kuhn et al. showed that the administration of amoxicillin for six months to five children diagnosed with ASD and Lyme disease improved their speech, eye contact, and sleep behaviors and led to a reduction in repetitive behaviors.^129^ A possible explanation for the differences in the findings of Sandler et al. and Kuhn et al. can be attributed to the selection of antibiotics, length of treatment and dosages, and differences in the assessment tools used to evaluate outcomes. Limitations to these studies include the sample size used for the trials, lack of control groups, lack of microbiota analysis, and the lack of prior knowledge on the mechanism of action of the selected antibiotics on the microbiota-gut-brain connection.

Vancomycin, a glycopeptide antibiotic primarily targeting gram-positive bacteria, can alter the gut microbiota significantly by affecting major bacterial phyla while sparing certain taxa linked to clinically relevant infections. In the seminal study by Sandler et al.,^55^ vancomycin showed temporary improvements in children with ASD, highlighting the potential role of gut microbial modulation. However, antibiotics alone cannot reverse dysbiosis or sustain improvements long term. Vancomycin has also been used as a pre-treatment for microbiota transplants^54^ with the hope that the benefits of vancomycin recorded by Sandler et al.^55^ are preserved while the disruption of indigenous microbiota by vancomycin enhances the engraftment of donor bacteria. Long-term use of vancomycin may induce undesirable shifts in microbial composition, such as an increase in Proteobacteria (gram-negative bacteria), potentially posing additional risks.

Future studies of antibiotics with a narrower spectrum that target primarily harmful bacteria with less effect on beneficial bacteria may be very useful. Also, research with randomized control trials, larger sample sizes, designed follow-up points, microbiota, and metabolites analyses might help identify the mechanism of action of specific antibiotics on the gut-brain axis and could provide physiological insights into their efficacy.

### Antifungals

In six studies, increased abundance of fungi or yeast (primarily *C. albicans*) has been reported in 25–58% of children with ASD at rates substantially higher than in typically developing controls.^10,25,34,130–132^ The overgrowth of fungi in ASD can be attributed to the increased use of antibiotics and lower bacterial diversity,^133^ which may allow opportunistic fungi/yeast like *Candida* to grow and dominate in the GI tract. *Candida* colonization is associated with inflammation and increased levels of inflammatory markers, such as cytokine IL-17.^134^ It has been reported that an increased abundance of *C. albicans* in ASD children correlates with a decreased abundance of beneficial bacteria *Faecalibacterium prausnitzii* and fiber consumer commensal *Prevotella copri*.^135^ Additionally, *C. albicans* have been observed with a bimodal distribution, and a higher relative abundance of *Candida* was associated with worse ASD symptoms.^136^ A national survey of over 27,000 autism families reported that two antifungals, Nystatin and Diflucan, had the highest reported benefit of any medication for ASD, with 62% and 55% reporting improvements in ASD symptoms and only 5% reporting worsening of symptoms.^128^ A small open-label clinical trial found that treating intestinal yeast overgrowth in children with ASD with antifungals led to some clinical improvements and reduced yeast metabolites.^137^ One of the limitations of these studies is the lack of data on the effect of the treatment on the bacterial community and the characterization of the fungal community to identify the changes in the fungal community. Data on long-term follow-up after treatment is also lacking in these studies. Overall, these studies suggest that intestinal fungi/yeast are common in children with ASD and may contribute to their ASD symptoms, and treatment with antifungals may be beneficial. However, future research with more rigorous antifungal studies is needed to determine which fungal species and subtypes of yeast or fungi are elevated in autism and how antifungal drugs can be used or modified to produce results that have a greater therapeutic action and also help to understand the mechanism of action of these antifungals. Also, future research should focus on the possibility of including dietary interventions to enhance the effect of antifungal drug therapy.

### Probiotics and synbiotics

Probiotics are microorganisms considered beneficial for gut health.^138^ Probiotics have emerged as a candidate microbiota-targeting intervention due to their safety and widespread acceptance.^139,140^ Synbiotics are probiotics combined with prebiotics;^141^ prebiotics are compounds that feed beneficial gut bacteria or probiotics. The results of clinical trials performed with probiotic and synbiotic supplementation interventions in ASD, that included microbiome analysis, are summarized in Table 1. The studies in the table are focused on trials that reported the effect on GI symptoms, ASD symptoms, and changes to gut microbiota communities. Also, all the studies reported in Table 1 are preliminary; they used different formulations of probiotic strains, synbiotics, and dosing regimens.

In a randomized, double-blind, placebo-controlled pilot trial, 35 individuals with ASD aged 3–20 years were randomly assigned to a treatment or placebo group. The trial consisted of two stages. In the first stage, the treatment group received the oral probiotic *L. plantarum* PS128 (probiotic only), while the placebo group received an oral placebo (placebo only) for 16 weeks. In the second stage, both groups received intranasal oxytocin (probiotic + intranasal oxytocin and placebo + intranasal oxytocin), and the trial proceeded for 28 weeks. The treatment group showed significantly more improvements in ASD symptoms after receiving *L. plantarum* PS128 and intranasal oxytocin compared to the placebo group. The authors also reported a greater increase in *Roseburia*, *Veillonella*, and *Streptococcus* bacteria.^142^ In another randomized double-blinded control trial carried out by Guidetti et al. consisting of 61 ASD participants (age 24 months–16 years), the authors reported improvement in GI symptoms, communication, and maladaptive behaviors, as well as an increase in beneficial bacteria, such as *Streptococcus thermophilus* and *Bifidobacterium longum*, and a decrease in species, such as *Alistipes finegoldii*, *Clostridium leptum*, and *Ruminococcus calldus* .^143^ It’s important to clarify that increased fecal abundance of some probiotic strains, like *Streptococcus thermophilus* and *Bifidobacterium longum*, come directly from the probiotic supplement and likely represent only a transient alteration in the native gut microbiota. However, a randomized control trial by Sherman et al. found no change in ASD symptom severity and GI symptoms but reported a significant increase in *Lactobacillus*.^144^ An open-label study by Shaaban et al. involved 30 ASD children found improvement in GI symptoms and ASD symptoms in addition to an increase in *Bifidobacterium* and *Lactobacillus* determined using quantitative PCR.^145^ Overall, these probiotic studies suggest several probiotic species may benefit ASD. However, most probiotics do not colonize and need to be taken daily.

Interventions with synbiotic supplementation are reported in Table 1. Li et al. recently published an open-label study consisting of 53 (age 3–12 years) children with ASD and 45 age-matched typically developing children.^146^ The authors reported significant improvement in ASD symptoms measured by Childhood Autism Rating Scale (CARS) scores and GI symptoms measured by Gastrointestinal Symptom Rating Scale (GSRS), and an increased abundance of *Bifidobacterium animalis*, *Akkermansia muciniphila*, and *Fusicatenibacter saccharivorans*, as well as a decrease in *Blautia obeum*. Another open-label synbiotic study by Phan et al. showed no improvement in the Social Responsiveness Scale (SRS2). However, there was an improvement in GI symptoms and increase in beneficial microbes, such as *Bifidobacterium* and *Lactobacillus* species.^57^ Wang et al. also observed a reduction in the severity of autism and improvements in GI symptoms compared to the placebo group, a significant increase in beneficial bacteria Bifidobacteriales and a decrease in *Clostridium* after probiotic intervention after synbiotic trial.^147^ Sanctuary et al. used *Bifidobacterium infantis* and colostrum, involving 8 ASD participants (2–11 years), and reported improvement in GI symptoms and aberrant behaviors.^148^ Arnold et al. also enrolled 10 ASD participants (3–12 years) and found improvement in GI symptoms and quality of life of the participants. Sanctuary et al. and Arnold et al. found no significant changes in the microbiome. A recently published case study by Aldegheri et al. about a 17-year-old male who received probiotics and 250 mg of human milk oligosaccharides (HMO) for 6 months saw improvement in behavior, such as aggressiveness and mood stability, and a decrease in the bacteria *Sutterella* species.^149^

Overall, these studies used different probiotics and synbiotics and generally found good tolerance and some benefit in GI and ASD symptoms compared to the baseline, with the randomized control trials (RCT) studies being more conclusive. In addition to improvement in ASD and GI symptoms, there was an increase in beneficial bacteria, such as *Bifidobacteria* and *Lactobacilli*, in some of the studies,^57,143,145–147^ which could potentially be attributed to their presence in the probiotic supplementation. Other genera, such as *Coprococcus* and *Roseburia*, were increased, and a decrease in bacteria, such as *Clostridium*, in some of the studies. One of the studies found an increase in specific bacteria, such as *Ligilactobacillus salivarius* and *Bifidobacterium longum*, which were part of the probiotics mixture used in the study.^143^

The current evidence for the efficacy of probiotics and synbiotics in ASD is inconsistent and complex because treatment across these studies is variable, with different formulations, dosages, lengths of treatment, and administration methods. Also, as seen in Table 1, only a few studies clearly stated their primary and secondary outcome measures,^57,142,145–147,150^ and only one study reported effect size^150^ after treatment. None of the studies investigated attempts to optimize dosage. Also, none of the studies presented in Table 1 involved post-treatment follow-up assessments, which makes it unclear if there were lasting benefits. However, it is likely that most of these effects were temporary and would require constant treatment to maintain benefits. Because different studies used different evaluation tools, which treatments were most/least effective is also unclear. To enhance improvement, future research should focus on normalizing ASD assessments for better inter-study comparisons, optimizing dosages, and identifying gut commensals linked to improved symptoms for developing future probiotics or microbial biomarker-based assessment metrics.

Some drawbacks of studies looking at probiotics/synbiotics as an intervention targeting the gut microbiota include the following: (1) It is unclear how one or a few bacterial strains can alter significantly dense gut microbial communities consisting of approximately 500 bacterial strains. Moreover, considering the microbiome variability among individual patients, it is difficult to expect consistent benefits. Microbial communities are very complex, and this complexity highlights the need for further research into the mechanisms through which probiotics influence microbial ecosystems and their potential to provide consistent therapeutic outcomes. (2) Long-term effects of repeated treatment on the gut microbiome are unknown. (3) the studies did not report on whether the content of the probiotics was verified to match the label prior to use in the clinical trials, which may be of concern when dealing with live bacteria, whose viability can be greatly affected by shipping and storage conditions. And (4) there was a lack of reports on participants who were unresponsive to the probiotics/synbiotics interventions; because most of these interventions are often supported by the manufacturers, and due to the industry interest, participants with negative results may remain unreported.

### Diet and prebiotics

Dietary interventions are major modulating factors of the intestinal microbiota,^151^ and dietary changes are commonly used for treating ASD-related symptoms.^152^ However, the many clinical trials of dietary interventions for ASD have generally not evaluated the effect on the microbiome, so we do not discuss them here. Yap et al. looked at the microbiome of 247 children, 99 with ASD, 51 related controls (siblings), and 97 unrelated controls.^153^ One of their conclusions was that dietary preferences drive differences between the gut microbiota of children with ASD and typically developing ones. This publication adds important knowledge concerning diet and the microbiome. However, we disagree with the main conclusion of this paper, which states that most or all the differences in microbiome composition reported previously are only because of diet and that there is no actual link between the gut microbiome and ASD. Diet is one of the factors that could differentiate the microbiome of ASD and TD due to the unique preferences in diets that are common in the ASD population; however, other factors, such as C-section birth, shorter duration of breastfeeding, excessive use of antibiotics,^39,40^ and host genetics,^42^ have also been identified to explain differences in the microbiome. A meta-analysis by Morton et al. showed that diet explained 3% of the variation in the microbiome of ASD and TD controls.^41^

Diet is known to affect the composition of gut microbiota. However, the responsiveness of individuals to different diets is expected to be heterogeneous, as how diets effect the microbiome depends on various factors, such as the existing composition of the microbiota and its metabolic activity, and extrinsic (lifestyle, medication) and intrinsic (immune and metabolic regulations) factors. Short-term dietary interventions can alter microbial diversity^154^; however, these alterations are transient, and whether long, prolonged dietary changes can induce permanent changes in the microbiota is unknown due to the lack of long-term follow-ups of short-term dietary interventions or lack of long-term human dietary interventions. Interventions with dietary approaches, such as organic substances (e.g., prebiotics), can selectively promote the metabolism and proliferation of beneficial microorganisms, thereby improving host health.

Regarding clinical trials of prebiotics in ASD described in Table 1, Inoue et al. found a significant reduction in constipation, behavioral irritability, and an increase in defecation per week, with a significant increase in *Blautia* and *Acidaminococcus* bacteria, while *Streptococcus*, *Odoribacter*, and *Eubacterium* decreased after treatment.^155^ A randomized, double-blind, placebo-controlled trial by Grimaldi et al. found that participants who received Bimuno galactooligosaccharide (B-GOS) treatment compared to placebo resulted in significant improvement in GI symptoms (abdominal pain and bowel movements), ASD symptoms (social behavior) and significant increase in beneficial microbes, such as *Coprococcus spp*., and *Dorea formicigenerans*.^156^ These two studies suggest that different prebiotics may benefit ASD, with the randomized control trial (RCT) on B-GOS being more conclusive. Despite these two studies demonstrating their benefits in improving ASD and GI symptoms, the results of their impact on specific microbes differed. This could be due to differences in the prebiotic products, heterogeneity in the ASD population, and their microbiota composition. As prebiotics are meant to enhance the proliferation of beneficial microbes, their impact on the microbiota also depends on the individual existing microbiota composition. This could also explain the differences observed in the impact of prebiotics on the microbiome.

### Fecal microbiota transplantation (FMT) and microbiota transplant therapy (MTT)

Fecal microbiota transplantation (FMT) is the most aggressive approach to modifying the microbiome. FMT is transplanted into patients to restore a healthy microbiota composition and function.^157,158^ FMT involves transplanting a consortium of gut microbiota from a well-screened healthy donor into a patient to modify their gut microbiota.^159^ Certain microbial species contribute to unfavorable gut microbiota and are linked to disease, and FMT aims to replace those unfavorable resident gut microbiota with favorable microbiota from a healthy donor. In brief, the preparation of FMT material typically involves screening donors for past and current GI issues, chronic illnesses, and infectious diseases, such as HIV and hepatitis. The microbiota can be delivered to the recipient through various techniques, such as enema, colonoscopy, nasal-gastric tubes, and orally (liquid, powder, or capsules).^160^ Currently, FMT is primarily used to treat *Clostridioides difficile* infections (CDI),^161–164^ where a patient is repeatedly treated with antibiotics to deplete their indigenous intestinal microbiota to clear the CDI infection. It mostly involves 1–2 administrations of intestinal microbiota from a healthy donor to restore the microbiota and offer resistance to CDI infection.^157,158,165,166^

Microbiota transplant therapy (MTT), a treatment derived from FMT, involves more intensive protocols to achieve donor engraftment and clinical benefit. Different elements of MTT use in ASD include pre-conditioning with antibiotics, bowel cleansing, and repeated administration of donor microbiota.^167,168^ *Microbiota* transfer *therapy* was used initially by Kang et al.^54^ However, this term is being replaced with *microbiota* transplant *therapy* as this term *transplant* recognizes the need for human donors and the engraftment of donor microbiota into recipients. From this point forward, we will refer to FMT studies that include antibiotic pre-treatment or multiple microbiota administrations as microbiota transplant therapy (MTT), rather than the general term FMT. Research into the effectiveness of MTT in treating individuals with ASD is still in its early stages. However, there is evidence that shows that MTT could aid in alleviating ASD and GI symptoms.

MTT studies for autism are summarized in Table 2. The first MTT study for ASD was an open-label clinical trial by Kang et al.^54^ with 18 children aged 7–16 years with ASD and gastrointestinal symptoms. The study found an initial 80% reduction in GI symptoms and a 23% improvement in ASD symptoms. These improvements were retained eight weeks after MTT treatment. A follow-up study at 2 years post-treatment found that most of the improvement in GI symptoms remained (59% reduction compared to baseline), and autism symptoms (Childhood Autism Rating Scale [CARS]) had continued to improve, with a 47% reduction compared to baseline.^169^ The study also found an increase in gut microbiota diversity, beneficial microbes (e.g., increased relative abundance of *Prevotella*, *Desulfovibrio*, and *Bifidobacteria*), functional genes, and a shift of microbial balance toward the microbiota composition of neurotypical children.^31,54,169^ The authors also showed a shift after MTT in plasma metabolite profiles, which became more similar to TD controls. In feces, levels of p-cresol sulfate decreased and became similar to levels in TD controls.^31,73,170,171^ This study demonstrated the potential of MTT as a therapeutic with long-term benefits for children with GI disorders.

Li et al. investigated the effects of a 4-week MTT protocol (administered 1×/week) with an eight-week follow-up involving 40 children with ASD (age 3–17 years) with GI symptoms and 16 age- and sex-matched TD children without GI problems in an open-label trial.^172^ Just as in Kang et al.^54^ the treatment involved a bowel cleanse where participants received polyethylene glycol before the donor microbiota capsules administration, but no antibiotics were supplied before treatment. Li et al. found that after MTT, children showed noticeable improvement in GI symptoms (35% decrease) and a 10% decrease in CARS scores after four weeks of treatment. They reported a shift in the bacterial community of ASD patients toward that of the TD controls, a significant decrease in *Eubacterium coprostanoligenes* bacteria abundance after MTT treatment, and a significant change in serum levels of neurotransmitters, such as GABA and 5-hydroxytryptamine (5-HT), which decreased after 4 weeks of MTT treatment but did not change further during the eighth week follow-up. GI and ASD improvements mostly continued at 8 weeks post-treatment, with some partial loss of benefit.^172^ The authors suggested that MTT may improve GI and ASD symptoms with extended treatment. A recently published study by Wang et al. also found significant improvement in GI and ASD symptoms in addition to a decrease in 5-hydroxyindoleacetic acid (5-HIAA) levels after MTT treatment.^173^

An open-label study by Li et al. investigated the effects of lyophilized donor microbiota (administered once every 4 weeks for 12 weeks) on 38 ASD children (age 3-14 years) with GI issues and 30 sex-matched TD children without GI problems – with an eight-week follow-up.^174^ There was no antibiotic treatment or a bowel cleanse before treatment. After MTT, children showed improvement in GI symptoms (51% decrease), a 10% decrease in CARS, and a 20% decrease in ABC scores. The authors reported a significant increase in *Eubacterium_hallii_group*, *Anaerostipes*, *Fusicatenibacter*, *Collinsella*, *Ruminococcus_torques_group*, and *Dorea*, and a decrease in the abundance of *Blautia*, *Prevotella*, and *Sellimonas*.

Chen et al. recently published an open-label study consisting of 29 ASD patients (aged 2–11 years) with GI symptoms and 36 TD children.^175^ The ASD children received two capsules of freeze-dried microbiota orally for 12 days per month for 4 months. The authors reported improved ABC, CARS, and GI symptoms and decreased *Collinsella* after treatment.

A case study by Hu et al. reported on a 7-year-old female with ASD treated with MTT.^176^ The child was pretreated with vancomycin antibiotic treatment for 2 weeks and a bowel cleanse prior to fecal donor microbiota (Table 2). The child received 80 ml of donor microbiota five times, each time separated by 1 week via colonoscopy. The core symptoms of ASD (assessed by ABC, CARS, SRS, ATEC) decreased after vancomycin treatment and further decreased after donor microbiota administration. The child’s gastrointestinal (GI) symptoms also significantly improved. After treatment, they also found that the child’s microbial diversity significantly increased with a significant increase in *Bacteroides* and *Ruminococcus* while *Bifidobacterium*, *Anaerostipes*, *Streptococcus*, and *Faecalibacterium* decreased. After treatment, the study showed a significant increase in SCFA, such as butyric acid.

Another case study was recently published by Hazan et al. about a 19-year-old male who received MTT from his typically developing female sibling.^177^ The patient received vancomycin treatment for ten days and a deep colonic wash prior to treatment, followed by a single dose of donor microbiota (300 mL) infused directly via colonoscopy (Table 2). After MTT treatment and during the follow-up, the patient experienced a significant improvement in behavioral and GI symptoms. The patient’s microbiome diversity also significantly increased after MTT, with an increase in *Bifidobacterium* and a decrease in *Lactobacillus animals* after treatment; these changes were maintained for at least during the 15-months.

The MTT studies described above suggest they can relieve not only gastrointestinal symptoms (e.g., constipation, diarrhea, indigestion, abdominal pain, and reflux) but also behavioral symptoms. However, these studies were open-label. Rigorous randomized, double-blind, placebo-controlled studies are needed to validate these findings. The Kang et al.^54,169^ studies showed short- and long-term improvements in ASD and GI symptoms, and Hazan et al. also found short and long-term benefits.^177^ The study by Li et al. found that benefits mostly continued at 8 weeks post-treatment but with some modest loss of benefit.^172^ A possible explanation for these different findings could be the different doses and treatment regimens used in each study (Table 2); for example, the Li et al. study did not involve pre-treatment with vancomycin,^172^ unlike the Kang et al.^54,169^ and Hazan et al.^177^ studies. The studies also showed that MTT changed intestinal microbiota composition. MTT led to an increase in microbial diversity,^31,54,169,176,177^ which is important because higher gut microbiota diversity is considered healthier in the context of the human gut. However, this is in contrast with Li et al., who did not find a significant change in microbial diversity.^172^ In addition to the increase in microbial diversity, Kang et al.^54,169^ and Hu et al.^176^ found that the treatment led to a shift in the microbial composition of the ASD participants to resemble that of the TD controls. Comparing a MTT study by Hazan et al., where a single dose of donor microbiota administration\was used,^177^ and Kang et al.,^54^ where multiple administrations of donor microbiota was used, changes in the microbiota were observed in both studies. However, the specific microbes that change in abundance differ, and Kang et al. reported a longer follow-up.

Variations in the findings of specific differences in the gut microbes after MTT treatment were also observed, which may be attributed to differences in donors, recipients, antibiotics use, cohort/geography, diet, treatment regimens, and donor microbiota preparation procedures. In the case of differences in the donors, studies have suggested that microbiome engraftment and clinical success depend on donor factors, such as the microbial composition of donor samples and successful engraftment of the donor microbes,^178,179^ and recipient factors, such as host genetics and diet. A recent paper by Chen et al. showed that a donor-recipient match indicated a likelihood of strain transfer and interactions between the donor and recipient microbes in ASD, which is important for species transfer and positive clinical outcomes in ASD.^175^ This evidence suggests the possibility of engraftment of donor microbiota as a factor that might have enhanced the treatment success for ASD. However, in the studies described above, engraftment of donor microbiota after MTT treatment were not reported. More precise engraftment measurements need to be incorporated into MTT investigations in ASD. Potential risks associated with MTT include the possibility of donors transferring opportunistic pathogens or multidrug-resistant organisms to recipients or causing infections. However, this can be avoided by carefully selecting and screening donors and their feces. Kang et al. found beneficial impacts of MTT continuing 2 years after completion of MTT treatment.^169^ However, more studies are needed to understand the long-term effects of MTT on ASD and the gut microbiome.

Furthermore, identifying an ideal “best personalized donor” whose microbiome produces metabolites tailored to meet the specific needs of a recipient based on the recipient health condition, genetics, existing microbiome composition, and donor-recipient compatibility could pave the way for MTT guided by precision medicine. Such an approach would enable interventions that are customized to individual differences, maximizing therapeutic benefits for ASD. Despite the promising effect of MTT intervention from these open-label studies, further research studies with randomized, double-blind, placebo-controlled studies are needed with a larger cohort to elucidate further their effect on ASD.^180–183^ Four randomized, double-blind, placebo-controlled clinical trials are expected to be completed in 2024 and 2025: (1) Trial no.: NCT03408886 and NCT04182633: these trials, expected to be completed in 2024, involve (a) adults (age 18–60 years) with ASD and GI issues and (b) children with ASD and GI issues. The treatment includes a 2-week vancomycin treatment, followed by 1-day bowel cleanse, 8-week MTT treatment for adults, and 12-week MTT treatment for children. Follow-up evaluations at 6, 12, and 18 months will be conducted. (2) Trial no.: ChiCTR2100043906 has started in China, involving 318 children (age 3–6 years) with ASD and GI issues, with results anticipated by the end of 2024. Participants will receive a 12-day treatment repeated every month for 4 months, followed by a 2-month follow-up.^184^ (3) Trial no.: ACTRN12622000015741 is expected to be completed by the end of 2025 in Australia. It includes 100 ASD adolescents and adults (aged 16–45 years) with mild to severe GI symptoms. The treatment involves an overnight bowel cleanse, 1–2 days of MTT treatment, and follow-up assessments at 6, 12, and 26 weeks post-treatment.^185^ (4) Trial no.: ChiCTR2200058459 involving 42 ASD children (age 3–9 years) in China. Treatment involves a bowel cleanse, a 12-week treatment period followed by a 12-week follow-up.^186^

Although research on MTT trials in ASD has been conducted, the available evidence is still insufficient, as all of the published studies to date are open-label.

**Figure 2.**
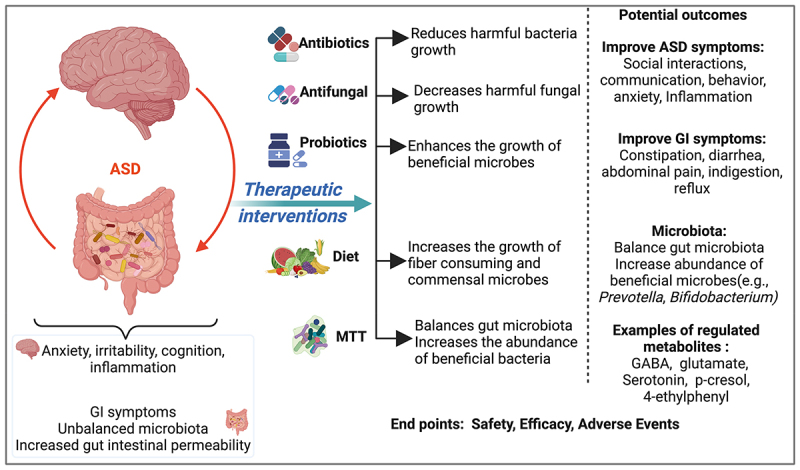
Summary of microbiota interventions targeting the gut microbiome and possibly GI and ASD symptoms outcomes.

The microbiota includes about several hundred bacterial species, as well as fungi and bacteriophages, and thus can reconstruct the gut microbial ecology,^187^ which regulates the bacterial community and also impacts the host immune system.^188^ As summarized in Figure 2, Findings from the reviewed studies suggest that interventions targeting the microbiome have some potential to address ASD symptoms and GI issues and have an effect on the microbiome composition in ASD. MTT seem the most promising of all the microbiome targeting interventions. MTT provide an entire set of gut microbiota from well-screened healthy donors. Probiotics may offer some health benefits but are limited to only introducing a small number of bacterial species most often cultured from non-human origins. Prebiotics may influence the growth of specific types of microbes, but some prebiotics may enhance the growth of harmful and beneficial bacteria. On the other hand, the disadvantages of the MTT approaches are the high costs of screening and production and the possibility of disease transmission with the transplanted material; the latter is minimized with careful screening.

Regarding safety and adverse effects, most intervention studies reported few to no adverse effects, suggesting that they may be safe and tolerable. For instance, MTT treatment has generally been associated with few adverse reactions, suggesting it is safe and well-tolerated in most cases. Kang et al.^54^ found that 5%, 39%, and 28% of participants suffered from mild to moderate rashes, hyperactivity, and tantrums/aggression, respectively, at the start of vancomycin pre-treatment, and lasting only a few days, but the microbiota was well-tolerated. In the study by Li et al.,^172^ short-term adverse effects were noted, including fever (3.7%), hyperactivity (11.4%), and tantrums/aggression (3.7%). In the probiotic studies, Shaaban et al. reported that all adverse effects were mild and transient during the probiotic intervention.^145^ In general, probiotics, prebiotics, synbiotics, and MTT appear relatively safe and seem to be a helpful approach for the alleviation of GI discomfort in children with ASD for the short and long term, in addition to shifting gut microbiota features toward that of neurotypicals and improving patient quality of life. However, antibiotics appear to have only short-term benefits, and overuse of antibiotics may have contributed to worse GI and ASD symptoms.

## Future perspectives of MTT studies in ASD

Emerging research into therapeutic interventions for the treatment of ASD symptoms is centered on microbial-based therapies, such as MTT, which have demonstrated potential in safely reducing ASD and GI symptoms and improving the microbiota. FMT therapy from a healthy donor to a patient is a proven treatment for *C. difficile*, and to enhance its efficacy, the American Gastroenterological Association (AGA) has developed guidelines.^189^ FMT is a well-established treatment for recurrent *C. difficile* infections (rCDI), often requiring just one or two doses to restore microbial balance. Given its success in reestablishing gut microbiota in rCDI, researchers have explored its potential in other conditions, including ASD. However, ASD is a more complex and clinically distinct condition with different microbiota alterations. As a result, this requires the use of MTT. MTT is a more sophisticated treatment that requires a pre-treatment with antibiotics, multiple doses and/or extended treatment durations to achieve lasting effects.

MTT hold promise as effective interventions for ASD. However, their application has many challenges, including donor selection criteria and factors, such as the microbial composition of donor samples, successful engraftment of the donor microbes, age of donor and donor-recipient compatibility, and host response.^175,190^ Other challenges include a lack of standardized assessment tools, comprehensive safety evaluations, an ideal dosing regimen, and a lack of standardization in the preparation and delivery.^191^

Some of the MTT studies described here did not include longer follow-ups. It is appropriate to incorporate longer follow-ups of clinical courses to further determine the interventions’ efficacy and safety. Further research and clinical studies are needed to identify the optimal treatment, dosing, and duration of MTT therapies, in addition to an in-depth understanding of the types of gut microbiota that may ameliorate ASD symptoms.

Future strategies to enhance the efficacy and long-term success of MTT in ASD treatment should focus on several key areas and for that, possible recommendations are: 1) Optimizing donor-recipient matching through microbiome-metabolomic profiling, host genetics, immune system compatibility, and machine learning methods may improve treatment outcomes by ensuring that the introduced microbial communities are more likely to engraft and persist. 2) Identifying reliable biomarkers for assessing intervention success is essential for tracking microbiota restoration and clinical improvements in ASD-related symptoms. 3) Incorporating dietary interventions or prebiotics alongside MTT may enhance the colonization and stability of beneficial microbes in the gut. Future trials should integrate dietary strategies to support sustained microbial and metabolic improvements before, during and after MTT administration. 4) Developing standardized ASD assessment tools is critical for ensuring consistency and comparability across clinical studies. The heterogeneity in ASD symptomatology underscores the need for validated, universally accepted outcome measures that accurately capture both GI and ASD symptoms improvement following MTT interventions. 5) Establishing standardized protocols for MTT preparation and administration in ASD-related research will enhance reproducibility and clinical translation. While some studies, such as Kang et al., have utilized standardized donor microbiota preparations under Good Manufacturing Practice (GMP) guidelines, the field would benefit from more comprehensive standardization, including dosage optimization, duration of treatment, and pre/post-treatment short and long-term monitoring. Establishing consensus guidelines will facilitate more rigorous clinical trial designs and help define best practices for future research in this area.

As MTT research in autism advances, it is essential to evaluate its safety and effectiveness thoroughly, and some of these trials are already underway.

## Summary

The microbiota-gut-brain axis is important in gastrointestinal symptoms and neurodevelopmental dysfunctions in ASD patients. Meta-analyses of gut microbiota in ASD have revealed substantial differences between ASD and TD children, with great heterogeneity in the ASD group. Antibiotic therapies may have only temporary effects, and overuse of antibiotics may have contributed to worse GI and ASD symptoms. In contrast, antifungals, probiotics, prebiotics, synbiotics, and MTT appear safe and possibly beneficial, with MTT leading to long-term improvements in some studies. More research is needed to determine if optimal probiotics, prebiotics, and synbiotics and their dosage can have better and longer-lasting effects on the gut microbiome and ASD symptoms. Randomized clinical trials of antifungals (with pre-screening to select participants with high levels of fungi) are needed to determine the effect of treatment on ASD-related symptoms. Future research is also needed on randomized double-blinded control trials of MTT to evaluate its safety and efficacy fully. Additionally, personalized treatment plans that consider individual microbial profiles, diet, and genetic backgrounds could enhance the effectiveness of these interventions. The potential of these interventions to offer a holistic approach to managing ASD symptoms underscores the importance of continued research and development in this field.

## Data Availability

Data sharing is not applicable for this study.
